# Magnetic Resonance Parkinsonism Index Is Associated with REM Sleep Behavior Disorder in Parkinson’s Disease

**DOI:** 10.3390/brainsci12020202

**Published:** 2022-01-31

**Authors:** Daniele Urso, Salvatore Nigro, Benedetta Tafuri, Valentina Gnoni, Marco Filardi, Roberto De Blasi, K. Ray Chaudhuri, Giancarlo Logroscino

**Affiliations:** 1 Center for Neurodegenerative Diseases and the Aging Brain, Department of Clinical Research in Neurology, University of Bari ‘Aldo Moro’, “Pia Fondazione Cardinale G. Panico”, Tricase, Lecce, Italy; daniele.urso@kcl.ac.uk (D.U.); salvatoreangelo.nigro@gmail.com (S.N.); benedetta.tafuri@gmail.com (B.T.); gnoni.vale@gmail.com (V.G.); marco.filardi1@gmail.com (M.F.); 2 Department of Neurosciences, Institute of Psychiatry, Psychology and Neuroscience, De Crespigny Park, King’s College London, London, UK; ray.chaudhuri@kcl.ac.uk; 3 Institute of Nanotechnology (NANOTEC), National Research Council, Lecce, Italy; 4 Department of Basic Medicine, Neuroscience, and Sense Organs, University of Bari ‘Aldo Moro’, Bari, Italy; 5 Department of Radiology, Pia Fondazione di Culto e Religione “Card. G. Panico”, Tricase, Italy; robertodeb55@gmail.com

**Keywords:** Parkinson’s disease, RBD, MRI

## Abstract

We investigated the association between the Magnetic Resonance Parkinsonism Index (MRPI) and REM sleep behavior disorder (RBD). We included 226 de novo PD patients (82 PD-RBD and 144 PD-noRBD) and 19 idiopathic RBD patients. Furthermore, 3T T1-weighted MR images were used for automated brainstem calculations. MRPI values were higher in the PD-RBD (*p* = 0.004) compared to PD-noRBD patients. Moreover, MRPI proved to be a significant predictor of REM Behavior Disorder Screening Questionnaire scores in PD (β = 0.195, *p* = 0.007) and iRBD patients (β = 0.582, *p* = 0.003). MRPI can be used as an imaging marker of RBD in patients with de novo PD and iRBD.

## 1. Introduction

REM sleep behavior disorder (RBD) is characterized by loss of physiological muscle atonia and episodes of dream-enactment behaviors during REM sleep [1]. RBD is associated with repeated injuries to the self and the bedpartner and with a major quality of life (QoL) burden [2]. Idiopathic RBD (iRBD) is now recognized as the prodromal stage of α-synucleinopathies, including Parkinson’s disease, which is by far the strongest prodromal marker [3]. Patients with PD and comorbid RBD have worse nonmotor symptoms, subjective motor performance, and QoL compared to PD patients without RBD [4]. Although RBD’s pathophysiology has not been completely elucidated, lesions in the brainstem structures, such as midbrain, pons, and medulla,- have been associated with RBD in both animal [5,6] and human studies [7].

Over the past years, several MRI techniques have been proposed to investigate neurodegeneration associated with RBD. White matter diffusivity alterations using MRI have been localized in the midbrain tegmentum, rostral pons, pedunculopontine nucleus, and pontine reticular formation in patients with iRBD [8,9]. Recently, microstructural changes have also been found in the superior and medial cerebellar peduncles [10,11] and distressing dreams have been shown to strongly correlate with superior cerebellar peduncle volume [12]. Recently, the Magnetic Resonance Parkinsonism Index (MRPI), which integrates the pons and midbrain areas and the width of middle and superior cerebellar peduncles, has been proven to be a measure to capture the midbrain and superior cerebellar peduncles atrophy, therefore characterizing patients with progressive supranuclear palsy [13,14]. In the present study, we investigated whether the MRPI could represent a useful neuroimaging marker able to capture the morphometric changes of brainstem subregions associated with RBD in de novo patients with PD and subjects with iRBD. 

## 2. Methods

### 2.1. Subjects

Data used in this article were obtained from the Parkinson Progression Marker Initiative (PPMI). The aims and methodology of the study have been published elsewhere and are available at https://www.ppmi-info.org/study-design/, accessed on 11 January 2021. Briefly, inclusion criteria for PD participants were the following: (1) aged > 30 years; (2) presence of two of the following: bradykinesia, rigidity, and resting tremor or an asymmetric resting tremor or asymmetric bradykinesia; (3) diagnosis made within the last 24 months; (4) PD drug naivety; and (5) dopamine transporter (DaT) deficit in the putamen on 123-I Ioflupane DaT imaging by central reading. Inclusion criteria for iRBD patients were: (1) aged ≥ 60 years and (2) confirmation of RBD by polysomnography (PSG) with central reading and/or clinical diagnosis of RBD by the site investigator, including existing PSG. The PPMI program was approved by the Institutional Review Board of each participating site. All participants to the PPMI gave their written informed consent to participate to the program.

### 2.2. Clinical Characteristics

Data extracted from the PPMI database included demographics, age at onset, disease duration, as well as clinical measures (such as Movement Disorder Society-Unified Parkinson’s Disease Rating Scale Part III (MDS-UPDRS-III) and Montreal Cognitive Assessment (MoCA)). The REM Behavior Disorder Screening Questionnaire (RBDSQ) was used to assess subjectively reported symptoms of RBD [15]. This 10-item structured questionnaire, which centers on the characteristics of dreams and dream-enactment behaviors, has been validated as a screening tool for RBD. A RBDSQ score of ≥ 5 demonstrated a sensitivity of 91% and a specificity of 77% [16]. PD patients with a RBDSQ score of ≥ 5 were classified as probable RBD (PD-RBD), while PD patients presenting a RBD score of < 5 were considered as without RBD (PD-noRBD). Furthermore, 123I-FP-CIT striatal binding ratios were available for PD subjects and were also extracted.

### 2.3. MRI Acquisition and Imaging Post-Processing

Data on non-contrast enhanced 3D volumetric T1-weighted brain 3 Tesla MRI scans were extracted. Details can be found at http://www.ppmi-info.org/wp-content/uploads/2010/07/Imaging-Manual.pdf, accessed on 11 January 2021. Brainstem regions involved in the MRPI (midbrain and pons area, middle and cerebellar peduncles width) calculation were automatically segmented on T1-weighted images using the automated approach described in previous studies [17]. Subsequently, automated segmentations were visually inspected and, when necessary, manual edits were performed to ensure proper measurement. Finally, MRPI values were computed by multiplying the ratio of the pons to midbrain area (P/M) by the ratio of the middle cerebellar peduncle to superior cerebellar peduncle width (MCP/SCP) [13,17].

### 2.4. Statistical Analyses

Between-group comparisons between PD-RBD and PD-noRBD subjects were performed by one-way ANCOVA or chi-squared test as appropriate. The associations between the RBDSQ score and MRI measures were explored using a multiple linear regression analysis with the RBDSQ as the dependent variable and MRI measures, age, sex, and disease duration as independent variables. The Benjamini–Hochberg False Discovery Rate (FDR) procedure was used to correct for multiple comparison. Statistical analysis was performed using Statistical Package for Social Sciences (SPSS 26.0) software (SPSS Inc., Chicago, IL, USA). A *p*-value of < 0.05 was considered statistically significant.

## 3. Results

A total of 82 PD patients were classified as PD-RBD while 144 were classified as PD-noRBD. Clinical data of the groups were compared and are illustrated in Table 1. There were no significant group differences in demographic characteristics (age and sex), motor burden (MDS-UPDRS), cognitive impairment (MoCA), and striatal binding ratio in the putamen and caudate (123I-FP-CIT). MRPI was higher in the PD-RBD group (*p* = 0.004) compared with the PD-noRBD group. The midbrain area was smaller in the PD-RBD group (*p* = 0.033) compared with the PD-noRBD group; however, there was no significant association after correction for multiple comparisons. No significant differences were found in the other morphometric measures. In the multiple linear regression model adjusted for age, sex, and disease duration, MRPI resulted a significant predictor of RBDSQ scores (β = 0.195, *p* = 0.007, Table 2). The midbrain area was not associated with RBDSQ score (β = −0.125, *p* = 0.089). No associations were found between other MRI measures and RBDSQ scores.

**Figure 1:**
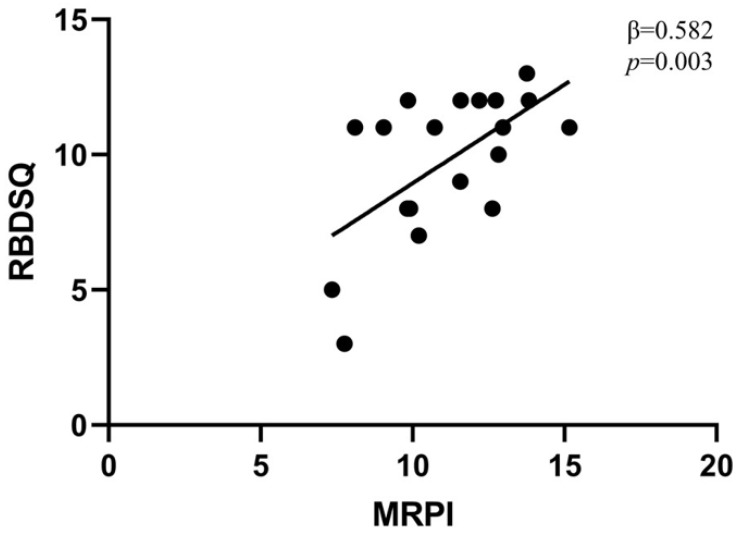
Association between MRPI and RBDSQ in subjects with idiopathic RBD. Scatter diagram illustrating the relationship between MRPI and RBDSQ in subjects with idiopathic RBD. MRPI, Magnetic resonance parkinsonism index; RBDSQ, REM Behavior Disorder Screening Questionnaire.

To substantiate our findings, we also explored the association between the RBDQS score and MRPI in the cohort of iRBD. The clinical characteristics of 19 iRBD patients are illustrated in Table 3. In the multiple linear regression model adjusted for age, sex, and RBD duration, MRPI was a significant predictor of RBDSQ scores (β = 0.582, *p* = 0.003, Figure 1). No other associations were found between MRI measures and RBDSQ scores.

## 4. Discussion

In this study, we found that PD patients with RBD had a higher MRPI than patients without RBD. Furthermore, MRPI values correlated with the RBDQS scores in both PD and iRBD patients. Overall, our findings show that combined MRI measures could be more helpful in disentangling the neurodegeneration pattern in RBD. Noteworthy, MRPI values were calculated automatically using an algorithm that can be easily implemented in clinical practice.

The precise network disrupted in RBD has not been entirely elucidated. However, evidence arising from animal and human studies has highlighted the involvement of neuronal structures localized to the brain areas of the dorsal midbrain and pontine tegmentum, the locus sub-coeruleus, and the medullary magnocellular reticular formation [18,19]. Previous neuroimaging studies in iRBD subjects have demonstrated microstructural alterations indexed by MRI diffusion parameters in brainstem regions [8,20]. *Scherfler* et al. localized significant decreases in fractional anisotropy in the tegmentum of the midbrain and rostral pons as well as increases in mean diffusivity within the pontine reticular formation overlapping with a cluster of decreased FA in the midbrain [8]. These findings were corroborated in a study on patients with PD and concomitant RBD, where RBD was associated with a prominent loss of volume in the ponto-mesencephalic tegmentum [21]. Interestingly, RBD has been recently linked to microstructural alteration in the cerebellar peduncles in patients with concomitant PD [10] and in iRBD [11]. In another study focusing on sleep disturbances, distressful dreams have been associated with white matter reduction in the superior cerebellar peduncle [12]. Although RBD was not specifically evaluated in that study, findings suggest that superior cerebellar peduncle may be involved in the genesis of RBD. Collectively, the changes observed through the brainstem so far indicate that RBD is implicated with a distributed structural and microstructural alterations, affecting multiple cell populations to differing degrees [21] and that a combined MRI measure could be more appropriate in unravelling abnormalities in an RBD structural brainstem network than single MRI measurements. Indeed, by adopting a combined MRI automatic measure that considers midbrain, pons areas, and width of superior peduncles, we found that MRPI values are able to discriminate between PD patients with and without RBD. Furthermore, MRPI correlates with RBDSQ, suggesting that an index combining several brainstem measures can be more useful in disentangling the network degeneration in RBD.

Some limitations should be recognized in this study. For instance, polysomnographic data were not available and could not be used to confirm the definitive diagnosis of RBD in the PD cohort. Second, the sample size of the iRBD cohort was relatively small. Third, although we found statistically different MRPI values between the groups, there is still a large overlap that does not allow to identify the presence of RBD at the individual level. Further studies with larger sample size are warranted to explore the associations between polysomnographic measures and MRPI.

## 5. Conclusions

In conclusion, automated MRPI, a combined brainstem measure, could represent a promising imaging marker of RBD in patients with de novo PD and iRBD.

## Figures and Tables

**Table 1:** Demographic and clinical differences in PD patients with and without RBD.

	PD-noRBD 999888(*n* = 144)	PD-RBD 999888(*n* = 82)	*p*
Age, years	60.73 ± 8.97	62.28 ± 9.76	0.364
Sex, male (%)	59.7	70.3	0.098
Disease Duration, months	7.17 ± 7.40	6.85 ± 6.68	0.752
MDS-UPDRS III	21.22 ± 9.10	22.18 ± 8.78	0.544
MoCA	27.35 ± 2.19	27.36 ± 2.08	0.698
Mean Putamen(123I-FP-CIT)	0.81 ± 0.26	0.83 ± 0.34	0.549
Mean Caudate(123I-FP-CIT)	2.00 ± 0.54	1.97 ± 0.62	0.978
Midbrain area	140.72 ± 43.29	127.79 ± 22.97	0.033
Pons area	514.49 ± 58.32	517.99 ± 76.45	0.926
MCP	9.12 ± 0.84	9.30 ± 0.83	0.195
SCP	3.95 ± 0.43	3.96 ± 0.46	0.753
Pons to midbrain ratio	0.76 ± 1.22	0.58 ± 1.08	0.274
MCP to SCP ratio	2.33 ± 0.32	2.37 ± 0.34	0.253
MRPI	8.89 ± 2.05	9.86 ± 2.20	0.004

Data are presented as mean ± SD or percentage. *Abbreviations:* MDS-UPDRS, Movement Disorders Society-Unified Parkinson’s Disease Rating Scale; MoCA, Montreal Cognitive Assessment; MCP, width of middle cerebellar peduncle; SCP, width of superior cerebellar peduncle; MRPI, Magnetic Resonance Parkinsonism Index, (P/M) × (MCP/SCP). Values in bold are corrected for multiple comparisons (FDR, q < 0.05).

**Table 2:** Coefficients of explanatory variables of RBD questionnaire scores in the multiple regression analysis in patient with PD.

Predictor	Standardized Regression Coefficient	*t*-Test	*p* Value
Age	−0.070	−0.981	0.327
Sex	−0.116	−1.758	0.080
Disease Duration	0.003	0.052	0.959
MRPI	0.195	2.736	0.007

Abbreviations: MRPI, Magnetic Resonance Parkinsonism Index.

**Table 3:** Demographic and clinical characteristics of subjects with idiopathic RBD.

	iRBD 999888(*n* = 19)
Age, years	70.35 ± 6.31
Sex, male (%)	89.4
RBD Duration, years	3.09 ± 3.61
MDS-UPDRS III	5.47 ± 3.89
MoCA	25.37 ± 4.53
Midbrain	117.89 ± 19.54
Pons	509.00 ± 38.11
MCP	9.03 ± 0.58
SCP	3.65 ± 0.68
M/P	0.23 ± 0.24
MCP/SCP	2.55 ± 0.50
MRPI	8.91 ± 1.81

Data are presented as mean ± SD or percentage. *Abbreviations:* MDS-UPDRS, Movement Disorders Society Unified Parkinson’s Disease Rating Scale; MoCA, Montreal Cognitive Assessment; MCP, width of middle cerebellar peduncle; SCP, width of superior cerebellar peduncle; MRPI, Magnetic Resonance Parkinsonism Index, (P/M) × (MCP/SCP).

## Data Availability

Data used in the preparation of this article were obtained from the Parkinson’s Progression Markers Initiative (PPMI) database (www.ppmi-info.org/access-data-specimens/download-data, accessed on 11 January 2021).

## References

[1] Boeve B.F., Silber M.H., Saper C.B., Ferman T.J., Dickson D.W., Parisi J.E., Benarroch E.E., Ahlskog J.E., Smith G., Caselli R.C. (2007). Pathophysiology of REM sleep behaviour disorder and relevance to neurodegenerative disease. Brain.

[2] Olson E.J., Boeve B.F., Silber M.H. (2000). Rapid eye movement sleep behaviour disorder: Demographic, clinical and laboratory findings in 93 cases. Brain.

[3] Högl B., Stefani A., Videnovic A. (2018). Idiopathic REM sleep behaviour disorder and neurodegeneration—An update. Nat. Rev. Neurol..

[4] Rolinski M., Szewczyk-Krolikowski K., Tomlinson P.R., Nithi K., Talbot K., Ben-Shlomo Y., Hu M.T. (2014). REM sleep behaviour disorder is associated with worse quality of life and other non-motor features in early Parkinson’s disease. J Neurol. Neurosurg. Psychiatry.

[5] Holmes C., Jones B. (1994). Importance of cholinergic, GABAergic, serotonergic and other neurons in the medial medullary reticular formation for sleep-wake states studied by cytotoxic lesions in the cat. Neuroscience.

[6] Webster H.H., Jones B.E. (1988). Neurotoxic lesions of the dorsolateral pontomesencephalic tegmentum-cholinergic cell area in the cat. II. Effects upon sleep-waking states. Brain Res..

[7] Iranzo A., Tolosa E., Gelpi E., Molinuevo J.L., Valldeoriola F., Serradell M., Sanchez-Valle R., Vilaseca I., Lomeña F., Vilas D. (2013). Neurodegenerative disease status and post-mortem pathology in idiopathic rapid-eye-movement sleep behaviour disorder: An observational cohort study. Lancet Neurol..

[8] Scherfler C., Frauscher B., Schocke M., Iranzo A., Gschliesser V., Seppi K., Santamaria J., Tolosa E., Högl B., Poewe W. (2011). White and gray matter abnormalities in idiopathic rapid eye movement sleep behavior disorder: A diffusion-tensor imaging and voxel-based morphometry study. Ann. Neurol..

[9] García-Lorenzo D., Santos C.L.-D., Ewenczyk C., Leu-Semenescu S., Gallea C., Quattrocchi G., Lobo P.P., Poupon C., Benali H., Arnulf I. (2013). The coeruleus/subcoeruleus complex in rapid eye movement sleep behaviour disorders in Parkinson’s disease. Brain.

[10] Ghazi Sherbaf F., Rahmani F., Jooyandeh S.M., Aarabi M.H. (2018). Microstructural changes in patients with Parkinson disease and REM sleep behavior disorder: Depressive symptoms versus non-depressed. Acta Neurol. Belg..

[11] Holtbernd F., Romanzetti S., Oertel W.H., Knake S., Sittig E., Heidbreder A., Maier A., Krahe J., Wojtala J., Dogan I. (2021). Convergent patterns of structural brain changes in rapid eye movement sleep behavior disorder and Parkinson’s disease on behalf of the German rapid eye movement sleep behavior disorder study group. Sleep.

[12] Radziunas A., Deltuva V.P., Tamasauskas A., Gleizniene R., Pranckevičienė A., Petrikonis K., Bunevicius A. (2018). Brain MRI morphometric analysis in Parkinson’s disease patients with sleep disturbances. BMC Neurol..

[13] Nigro S., Antonini A., Vaillancourt D.E., Seppi K., Ceravolo R., Strafella A.P., Augimeri A., Quattrone A., Morelli M., Weis L. (2020). Automated MRI Classification in Progressive Supranuclear Palsy: A Large International Cohort Study. Mov. Disord..

[14] Quattrone A., Nicoletti G., Messina D., Fera F., Condino F., Pugliese P., Lanza P., Barone P., Morgante L., Zappia M. (2008). MR Imaging Index for Differentiation of Progressive Supranuclear Palsy from Parkinson Disease and the Parkinson Variant of Multiple System Atrophy. Radiology.

[15] Stiasny-Kolster K., Mayer G., Schäfer S., Möller J.C., Heinzel-Gutenbrunner M., Oertel W.H. (2007). The REM sleep behavior disorder screening questionnaire-A new diagnostic instrument. Mov. Disord..

[16] Li K., Li S.-H., Su W., Chen H.-B. (2017). Diagnostic accuracy of REM sleep behaviour disorder screening questionnaire: A meta-analysis. Neurol. Sci..

[17] Nigro S., Arabia G., Antonini A., Weis L., Marcante A., Tessitore A., Cirillo M., Tedeschi G., Zanigni S., Calandra-Buonaura G. (2016). Magnetic Resonance Parkinsonism Index: Diagnostic accuracy of a fully automated algorithm in comparison with the manual measurement in a large Italian multicentre study in patients with progressive supranuclear palsy. Eur. Radiol..

[18] Boeve B.F. (2010). REM sleep behavior disorder: Updated review of the core features, the REM sleep behavior disorder-neurodegenerative disease association, evolving concepts, controversies, and future directions. Ann. N. Y. Acad. Sci..

[19] Lu J., Sherman D.M., Devor M., Saper C.B. (2006). A putative flip–flop switch for control of REM sleep. Nature.

[20] Unger M., Belke M., Menzler K., Heverhagen J., Keil B., Stiasny-Kolster K., Rosenow F., Diederich N.J., Mayer G., Möller J.C. (2010). Diffusion Tensor Imaging in Idiopathic REM Sleep Behavior Disorder Reveals Microstructural Changes in the Brainstem, Substantia Nigra, Olfactory Region, and Other Brain Regions. Sleep.

[21] Boucetta S., Salimi A., Dadar M., Jones B.E., Collins D.L., Vu T.T.D. (2016). Structural Brain Alterations Associated with Rapid Eye Movement Sleep Behavior Disorder in Parkinson’s Disease. Sci. Rep..

